# Anti-Proliferative and Apoptotic Activities of *Rumex crispus*

**DOI:** 10.3390/life14010008

**Published:** 2023-12-20

**Authors:** Sepideh Mohammadhosseinpour, Mukund Bhandari, Dallas A. Lee, Beatrice Clack

**Affiliations:** 1 Department of Biotechnology, Stephen F. Austin State University, The UT System, Nacogdoches, TX 75965, USAdal83@txstate.edu (D.A.L.); bclack@sfasu.edu (B.C.); 2 Molecular Biosciences Graduate Program, Arkansas State University, Jonesboro, AR 72401, USA; 3 Greehey Children Cancer Research Institute, UT Health, San Antonio, TX 78229, USA; 4 MSEC Program, Texas State University, San Marcos, TX 78666, USA

**Keywords:** *Rumex crispus*, natural product, DLD-1 cells, apoptosis, caspases

## Abstract

Colorectal cancer is the fourth leading cause of cancer death and the third most common cancer diagnosed in the United States. Several anticancer compounds from natural products have been of great interest in cancer chemotherapy and are currently in clinical trials. Natural products that present the targeted killing of cancerous cell and are soluble in water with minimal side effects are ideal candidates. In this study, water-soluble compounds from *Rumex crispus* plants were screened for anti-proliferative and apoptotic activity against human colorectal adenocarcinoma (DLD-1) cells. The most potent fraction with the highest cell killing and caspase fold change rates was selected for further experiments. The observed changes were further validated by measuring the caspase fold change using RT-qPCR. Furthermore, gene transcript levels were evaluated using an RT2 Profiler assay and a microarray experiment. Our results showed that the most potent L19 fraction exhibits anti-proliferative activity in a dose-dependent manner. The L19 fraction was found to induce apoptotic pathways by triggering different caspases and inflammatory pathways through the activation of non-apoptotic genes. Our study identified and validated the anticancer property of the L19 fraction, which can serve as a strong lead compound for the synthesis of other novel potent analogues.

## 1. Introduction

Colorectal cancer is the fourth leading cause of cancer death in the United States, killing around 50,000 people per year as of 2022 [1] (Supplementary Figure S1a). According to the National Cancer Institute’s Surveillance, Epidemiology, and End Results Program (SEER) database, colorectal cancer is also the third most common cancer diagnosed in the United States, with approximately 153,020 new cases and 52,550 deaths estimated in the year 2023 [2]. Due to the ongoing awareness of risk factors and colorectal cancer screening tests, early detection and surgical removal of colorectal polyps remain standard practices, helping doctors decrease the incidence rates of cancer development. Statistics show that, during the years 2013–2017, the incidence rates of colorectal cancer declined by 1% each year among adults aged 50 years and older but have increased by 2% per year among adults younger than 50 years of age [1] (Supplementary Figure S1b). Mainly, colorectal cancers develop through a series of genetic and epigenetic changes from benign polyps [3]. Some of the identified major hallmarks of cancer are resistance to apoptosis, replicative immortality, activating invasion, and metastasis [4]. Therefore, a better understanding of the apoptosis mechanism is crucial for the development of effective anticancer treatment and therapies [5].

Natural products have been of great interest in the canonical development of cancer chemotherapy because they provide foundational chemical leads of core moieties for the construction of novel compounds with enhanced anticancer properties [6,7,8]. Clinically useful anticancer compounds derived from natural products include vincristine, vinblastine, camptothecin derivatives, topotecan and irinotecan, etoposide, epipodophyllotoxin, and paclitaxel (Taxol) [9,10], and several other compounds, such as quercetin and resveratrol, are in investigational multi-drug cocktail clinical trials aimed at providing more effective treatments for cancer types that are chemically resistant to mono-therapeutic treatments [11]. The induction of apoptosis is critical for screening targeted anticancer properties from novel chemotherapeutics. Apoptosis is a type of cell death that is characterized by the activation of cellular caspase proteins that start a cascade of protein proteolyzation [12] when activated either via an intrinsic (mitochondrial) cell pathway or an extrinsic (death-receptor) cell pathway [13].

*Rumex crispus*, also known as Curly Dock or Yellow Dock, belongs to the family Polygonaceae and is historically documented in the treatment of cancerous lesions in Native American medicine [14]. So far, 29 Rumex species have been studied, and 268 compounds have been reported, including quinones, flavonoids, tannins, stilbenes, naphthalenes, terpenes, diterpene alkaloids, lignans, and other classes of phytochemical compounds [15]. Traditional chemical processing of *Rumex crispus*, with organic solvents petroleum ether and ethyl acetate, has identified β-Sitosterol, hexadecanoic acid, hexadecanoic-2,3-dihydroxy propyleste, chrysophanol, physcion, emodin, chrysophanol-8-O-beta- D-glucopyranoside, physcion-8-O-beta-D lucopyranoside, emodin-8-O-beta-D-glucopyranoside, gallic acid, (+)-catechin, kaempferol, quercetin, kaempferol-3-O-alpha-L-rhamnopyranoside, and quercetin-3-O-alpha-L-rhamnopyranoside [16]. Previous studies have shown the variability of chemical constituents of individual plants reported as isolated phytochemicals of hexacosanoic acid, β-Sitosterol, Epicatechin, Catechin, Kaempferol-3-O-α-L-rhamnoside, Kaempferol, 1,5-Dihydroxyanthraquinone, Rumexone, Emodin-8-O-β-D-glucoside (PMEG), and Emodin (1,6,8-trihydroxy-3-methylanthraquinone) in *Rumex crispus* [15]. These pharmacologically active classes of compounds, such as the anthraquinones (e.g., emodin) [17], flavonoids (e.g., quercitrin) [18,19], and tannins [20] contained in *Rumex crispus*, have shown anticancer properties and provide new pathways for researching less toxic, anticancer natural products [21]. *Rumex crispus* extracts have demonstrated induction of apoptotic signaling in different cancer cell lines [22]. Previously, we have also shown that *Rumex crispus* possesses anticancer properties against DLD-1 colorectal cancer cells [23,24] and that water-soluble extracts are more potent than extracts obtained from organic solvents. The identification of new compounds able to effectively kill cancerous cells that are both water-soluble and demonstrate minimal side effects is crucial [25], and said compounds would be ideal candidates for drug development. The main objectives of this study are to extract water-soluble compounds from the tissues of the *R. crispus* plant via aqueous accelerated solvent extraction, screen the constituent fractions separated using HPLC for cell viability, test the phytochemical fractions for anticancer properties in DLD-1 colorectal cancer cells, investigate the induction of apoptosis with a caspase assays, and measure the changes in apoptotic gene transcript levels through mRNA transcripts.

## 2. Materials and Methods

### 2.1. Rumex crispus Collection

**Figure 1:**
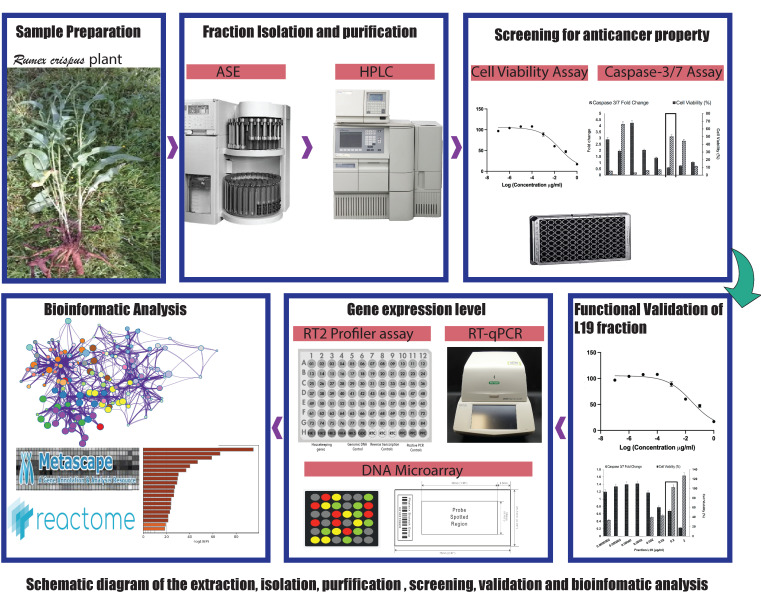
Schematic workflow of the extraction, isolation, purification, screening, validation of the L19 fraction from the *Rumex crispus* plant, and bioinformatics analysis of the gene transcription data. The figure depicts the workflow from the collection of the samples from the plant’s natural habitat to assessing its cell-killing properties using different biochemical assays.

Mature seeds bearing *Rumex crispus* plants were collected from Nacogdoches, TX, USA (31.6024865 N, 94.5677874 W), in April 2014. The mature *R. crispus* plant is shown in Supplementary Figure S2. Only the root and leaf sections of the *R. crispus* plant were used; the seed-bearing parts, along with the stems, were discarded. The leaves and roots were collected separately in different plastic bags. The roots were washed with water and air-dried for 10 min. The root and leaf samples were separately ground using a Waring Laboratory CB15 food blender (Waring, McConnellsburg, PA, USA) and liquid N2 to pulverize them into a powder. The ground plant tissues were stored at −80 °C for further use and analysis. Further steps were performed as shown in the schematic flowchart (Figure 1).

### 2.2. Accelerated Solvent Extraction

The frozen-powdered plant sample (3 g) was mixed with equal parts sand (Sigma-Aldrich, St. Louis, MO, USA) and loaded into an ASE 11 mL stainless steel vessel according to the manufacturer’s instructions for the Dionex Model ASE200 (Dionex Corp., Sunnyvale, CA, USA) accelerated solvent extraction system. ASE was performed at 85 °C heating at 1500 psi for 5 min for 3× cycles, with an elution volume of 35 mL. Deionized water (dH_2_O) was used as the polar extraction solvent to maximize the likelihood of aqueous soluble phytochemicals, and compressed laboratory-grade nitrogen was used to pressurize the system. The plant extract solutions were transferred to 50 mL polypropylene conical tubes and frozen at −80 °C before freeze-drying using a LABCONCO FreeZone 6 Liter freeze-dry system (Kansas City, MO, USA). Each ASE tube produced approximately 70–100 mg of dried material.

### 2.3. High-Performance Liquid Chromatography

The lyophilized extracts from ASE were used to prepare the samples for high-performance liquid chromatography (HPLC). Twenty-five milligrams of lyophilized ASE extract were weighed and resuspended in a 1 mL solution containing 95% *w*/*v* acidified (H_3_PO_4_) dH_2_O and 5% acetonitrile. The extract solution was filtered and loaded in HPLC sample vials. Acidified dH_2_O (pH 2.2) with HPLC-grade phosphoric acid was used as solvent A, and HPLC-grade acetonitrile (ACN) was used as solvent B. A total of 100 µL of the samples was injected onto a Zorbax Eclipse Plus 18 3.5 μm 4.6 × 100 mm (Aglient, Santa Clara, CA, USA) column with an AplhaBond C18 10 μm guard column (Supelco, Sigma-Aldrich, St. Louis, MO, USA) attached to a Waters 2695 HPLC Separations Module with Waters 2487 Dual λ Absorbance Detector (Waters Corp., Milford, MA, USA) for the separation and detection of the fraction peaks at 210 nm. An increasing gradient concentration of ACN from 5% to 45% for 60 min and isocratic concentration of 95% ACN for 10 min was used for elution with a 1 mL/min flow rate. A Spectra/Chrom CF-1 Fraction Collector (Spectrum Chromatography, Houston, TX, USA) was used to collect 1 mL fractions in a 1.5 mL microfuge tube every minute. Data were recorded using a NI USB-6221 data acquisition module and the NI LabVIEW Signal Express 2.5.1 software (National Instruments Corp., Austin, TX, USA). All fractions were collected and screened to investigate their apoptotic and anti-proliferative activities. Once the most potent fraction was confirmed, subsequent HPLC runs only collected the fractions with (+) experimental results assessed via DLD-1 viability assays.

### 2.4. Cell Culture and Cell Viability Assay

Human colorectal adenocarcinoma cells, DLD-1 cells (ATCC^®^ CCL-221™), were cultured in RPMI 1640 Media (Gibco^®^ Thermo Fisher Scientific, Waltham, MA, USA & Sigma-Aldrich, St. Louis, MO, USA) containing L-Glutamine supplemented with 10% (*v*/*v*) fetal bovine serum (FBS) (Atlanta Biologicals, Norcross, GA, USA), and 5 mL of a 100× Antibiotic-Antimitotic (Gibco^®^) was used as the cell culture medium in 6% CO_2_ at 37 °C. The DLD-1 cells were chosen as they are the most commonly used cell line in colorectal cancer research. The representative Brightfield image of DLD-1 cells at 200 × magnification and ISO1600 is shown in Supplementary Figure S3. The medium was changed every two to three days as indicated by the pH indicator phenol red. The cells were harvested with 0.25% Typsin and centrifuged at 2000 rpm and collected. The cell counts were measured with a Neubauer-Improved hemocytometer. Cell viability was measured using an MTS assay, a tetrazolium compound (Owen’s reagent) producing a colored formazan product converted by NADPH or NADH production. The cancer cells (1 × 10^4^ DLD-1 cells in 100 μL/well) were cultured in 96-well plates with serum-free media and treated with 100 μL of the sample extracts resuspended in RPM 1640 media (Gibco^®^ Thermo Fisher Scientific, Waltham, MA, USA & Sigma-Aldrich, St. Louis, MO, USA). An initial screen of all the HPLC fractions was tested for cell viability activity, and, later, the most potent fraction was chosen for additional validation testing. Untreated cells with an additional 100 μL of RPMI 1640 media were used as the negative control, and 100 μL of 50 µM Doxorubicin HCl in RPMI 1640 media was used as the positive control to measure both cell viability and apoptosis. The cells were cultured in microtiter 96-well plates incubated at 6% CO_2_ at 37 °C for 24 h. After 24 h of incubation with fractions (or control), the media were replaced with 100 μL of fresh RPMI 1640 media. Twenty microliters of CellTiter 96 AQueous One Solution Cell Proliferation Assay (Promega Corp., Madison, WI, USA) was added in each well and mixed using a VersaMax Microplate Reader (Molecular Devices, LLC, Sunnyvale, CA, USA). The culture plate was incubated in 6% CO_2_ at 37 °C for 4 h according to the manufacturer’s technical bulletin. Absorbance was measured at 490 nm using a VersaMax Microplate Reader with the SoftMax Pro 5.2 Software (Molecular Devices, LLC, Sunnyvale, CA, USA). The percentage of cell viability (%C_v_) of the DLD-1 cells as a measure of cell death was calculated using the absorbance of the treated cells (A_T_) relative to the absorbance of the untreated cells (A_UT_), multiplied by 100.

### 2.5. Apoptosis Assay

Screening cell death for the apoptotic signal caspase-3/7 activation assay was performed using 100 μL of the *R. crispus* HPLC fractions resuspended in RPMI media to investigate the relative caspase-3/7 activity in DLD-1 cells (200 μL total volume/well). The DLD-1 cells (1 × 10^4^ cells/100 μL/well) were subsequently cultured in a 96-well culture plate, using a multichannel pipette, with serum-free media and treated with 100 μL of the sample extracts resuspended in RPMI media. Initially, all HPLC fractions were tested for caspase-3/7 activity to determine potency and screen-out negative results. The apoptotic controls consisted of untreated DLD-1 cells with an additional 100 μL of RPMI media as the negative control and 100 μL of 50 µM Doxorubicin HCl (DOX) in RPMI media was used as the positive control. The assays were incubated at 6% CO_2_ at 37 °C for 24 h. After 24 h of incubation, the media containing *R. crispus* extracts were removed and replaced with 25 μL of fresh RPMI media. Apoptosis assays consisting of 25 μL of Apo-ONE Homogeneous caspase-3/7 (Promega Corp., Madison, WI, USA) were prepared following the manufacturer’s instructions, subsequently mixed using the shaking function of a VersaMax Microplate Reader (Molecular Devices, LLC, Sunnyvale, CA, USA), and incubated in the dark at room temperature for 1 h. The imaging and data of the plates were collected on a Typhoon Trio plus imager (GE Healthcare Life Sciences, Pittsburgh, PA, USA) (Ex: 499 nm, Em: 521 nm + 3 platen). The data were analyzed using ImageQuantTL v1.0 (GE Healthcare Life Sciences, Pittsburgh, PA, USA) to obtain relative fluorescence units (RFU) based on the controls. The fold change was calculated using the RFU of the treated cells (R_t_) relative to the untreated cells (R_ut_) and normalized with the percentage of cell viability for the respective fractions. Equation (1) was used in the calculation of the fold change.

Fold Change = (R_t_/cell viability of treated cell)/(R_ut_/cell viability of untreated cell) (1)

### 2.6. Real-Time Quantitative Polymerase Chain Reaction (RT-qPCR)

To assess the transcript levels of apoptotic genes, human colorectal adenocarcinoma cells, DLD-1 cells, were incubated with the most effective fraction, L19, at 2 µg/mL, in 6% CO_2_ at 37 °C. The total RNA was extracted from the L19-treated and -untreated DLD-1 cells at time intervals of 8 and 12 h using TRizol Plus RNA purification kits (Ambion Corp., Austin, TX, USA) following the manufacturer’s instructions (Supplementary Figure S4). The quantified RNA was reverse-transcribed using GoScript^TM^ reverse transcriptase (Promega Corp., Madison, WI, USA). The cDNA was purified using the Wizard SV Gel and PCR Clean Up System (Promega Corp., Madison, WI, USA) and quantified using a nanodrop cuvette. The fold change in the gene transcripts of CASP 1, 3, 4, 5, 6, 7, 8, 9, and 10 in the treated and untreated cells was assessed with a BioRad CFX96 Real-Time PCR System. Glyceraldehyde-3-Phosphate Dehydrogenase (*GAPDH*) was used as the housekeeping gene, and a 50 µM DOX treatment was to serve as a control for apoptosis. Caspase forward and reverse primers are listed in Supplementary Table S1. The RT-qPCR conditions were run with an initial denaturation at 95 °C for 10 min, followed by 40 cycles of denaturation at 95 °C for 30 s, with an annealing temperature at 60 °C for 60 s and an extension temperature at 72 °C for 30 s. The RT-qPCR resulted in Ct (cycle threshold) values for each gene, measured in triplicates. The ΔΔCt values were determined by calculating the difference between the ΔCt Treated and ΔCt Untreated measures. ΔCt Untreated was obtained by calculating the difference between the Ct Untreated and Ct GAPDH measures. Similarly, ΔCt Treated was calculated using the difference between the Ct Treated and Ct GAPDH Treated measures. GAPDH was used as the housekeeping gene.

### 2.7. RT^2^ Profiler Human Apoptosis PCR Array

The relative transcripts of the apoptotic genes involved in apoptosis were analyzed following the L19 fraction treatment of DLD-1 cells using an RT^2^ Profiler Human Apoptosis PCR array (Cat no: PAHS-012ZA, SA Biosciences, Qiagen, CA, USA) in adherence to manufacturer’s instructions. Briefly, DLD-1 cells treated with or without the L19 fraction (2 µg/mL) were incubated at 37 °C under a humidified atmosphere containing 6% CO_2_ for three different time points—6 h, 8 h, and 12 h. Gene-specific primer pairs were used in the 96-tube plate to which 10 µL of 2X SYBR green master mix (Promega Corp., Madison, WI, USA) and 20 ng cDNA template buffer- and nuclease-free water were added to obtain 20 µL of final reaction volume. The cDNA for both the untreated or treated samples was prepared as a master mix and distributed into the 96 tubes within the plate. The plates were sealed with a thin plastic film and centrifuged at 300× *g* for 5 min to remove bubbles, then placed into a BioRad CFX96 Real-Time PCR thermocycler following the same procedure as for the qRT-PCR. One 96-tube plate was used for the cDNA isolated from the treated cells, and another 96-well plate was used for the cDNA isolated from the untreated cells for each respective time point. The RT^2^ plate contained 84 apoptotic and non-apoptotic genes translation probes and controls, which included 47 pro-apoptosis genes, 21 anti-apoptosis genes, and 15 genes encoding regulatory proteins (which may either trigger or suppress apoptosis); details of the array are listed in the manufacturer’s catalog (Cat no: PAHS-01Z). Changes in mRNA transcription were analyzed using the ΔΔCt method, as described earlier in Section 2.6. The mRNA transcription levels were quantified relative to the values obtained for two human housekeeping (HK) genes—glyceraldehyde-3-phosphate dehydrogenase (*GAPDH*) and β-actin (*ACTB*).

### 2.8. Microarray Analysis

For the microarray analysis, transcripts of apoptotic genes were reverse-transcribed with SuperScript Plus Indirect cDNA Labeling System (Invitrogen, Waltham, MA, USA) according to the manufacturer’s instructions. Reactions utilized 5 µg of the total RNA with RT Reaction Mix, gently mixed it with the annealing mix, and then heated it at 46 °C for 2–3 h. The cDNA was purified using the Wizard SV Gel and PCR Cleanup System (Promega Corp., Madison, WI, USA), as described previously. Fluorescent labelling of cDNA was carried out using amino reactive Alexa Fluor (Em550 nm) and Alexa Fluor (Em647 nm) (ThermoFisher Scientific Corp., Grand Island, NY, USA). The amino allyl-labeled cDNA from both the untreated and treated cells was purified from the unreacted dye and buffer using a Wizard SV Gel and PCR Cleanup System according to the manufacturer’s protocol (Promega Corp., Madison, WI, USA), and the amount of cDNA (ng) and dye incorporated was calculated using the manufacturer’s instructions. The microarray assay was performed as a dye swap assay using the Human OneArrayv7 (OneArray Corp., San Diego, CA, USA). Hybridization was performed according to the manufacturer’s instructions.

The arrays were scanned at a 10-micron resolution setting with the Cy3 setting (555 nm) on channel 1 and the Cy5 setting (647 nm) on channel 2 using a Typhoon FLA 9500 (GE Health Science, Pittsburgh, PA, USA) scanner for the image acquisition and data analysis. The images were saved in a 16-bit TIF file format for processing. The quantification of the resulting data was analyzed using SpotXel 1.1 (Sicasys Software for Life Sciences, Heidelberg, Germany) to align the grid obtained from the array manufacturer using the .gal file provided and calibrated to the control corner spots on the array. The spot intensities were quantified for both the green and red channels, and the background was subtracted to provide the corrected spot intensities as output .gpr files. The noise data were processed using 50% of the spot size, and a value of 30 was selected for the largest size defect. The data resulting from SpotXel were converted to a .mev (MultiExperiment Viewer) file using Express Converter version 2.1 for use in MIDAS version 2.22-b0 (Microarray Data Analysis System). Normalization of the microarray data was performed using MIDAS to normalize the intensities within an array as well as between the dye swap pairs of arrays and to remove the bias attributable to the different dye intensities. The total intensity was selected with Cy3 chosen as a reference signal. A Lowess fit was selected along with the default parameters for iterative log mean-centering normalization and cross-log ratio data keep range. BRB-ARAY tools were used to obtain the average change in the gene transcription levels and obtain p-values for the respective data between gene families.

## 3. Results

### 3.1. Aqueous Soluble Compounds from Rumex crispus

The harvesting of wild *Rumex crispus* plants was chosen out of the abundant local populations of the plant in the nemoral iron-rich sandy clay soils of East Texas. Supplementary Figure S2 shows a photograph of a freshly harvested mature *Rumex crispus* plant from the sample collection site. After the collection and processing of the samples in the laboratory, samples of the root and leaf were isolated and prepared with ASE to produce ~70–100 mg of dried aqueous soluble phytochemicals. The extracted samples from ASE were further separated and fractionated using HPLC. In order to obtain enough material for repeatable cell assays, the experiments were repeated.

### 3.2. HPLC Fractions for DLD-1 Cell Viability and Caspase-3/7 Expression Activity

**Figure 2:**
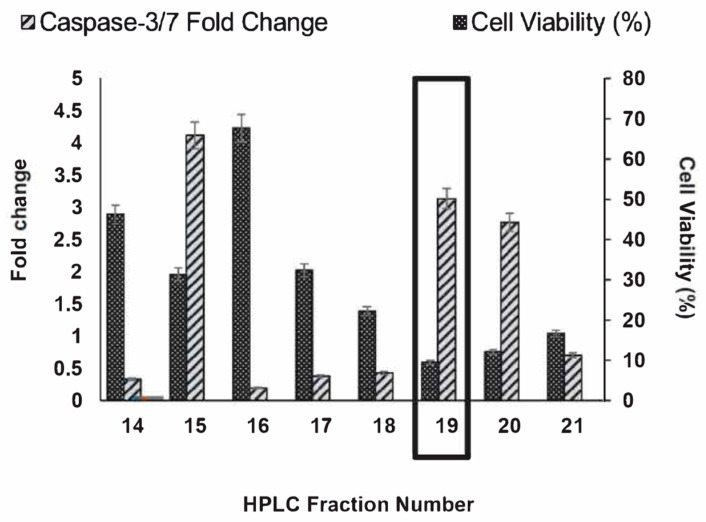
Cell viability percentage and caspase-3/7 fold change in the DLD-1 cell line for HPLC leaf fractions L14-L21. The cell viability was assessed using an MTS assay and by measuring the A490. Both the caspase-3/7 fold change (left axis) and the percentage of cell viability (right axis) were normalized to the untreated cell controls (n = 3) for each fraction.

The initial screening of HPLC fractions in DLD-1 cell cultures from both *R. crispus* leaf and root tissues showed fraction L19, isolated from the leaves, as the most potent fraction, with the highest inhibition of cell proliferation, as shown in Supplementary Figures S5 and S6. Additional confirmation of the most potent L19 compound was carried out in triplicate with isolated fractions of L19 and other fractions closer to L19, demonstrating that these fractions have similar biochemical properties. In addition to the positive confirmation of the cell killing assay and caspase activity assay, L19 also showed up as the most potent fraction, with less than 10% cell viability and more than a three-fold change in caspase-3/7 activity when compared to the controls (Figure 2). The fraction L19 was selected for further experiments.

### 3.3. Dose-Dependent Behavior of L19 Fraction on Cell Viability

**Figure 3:**
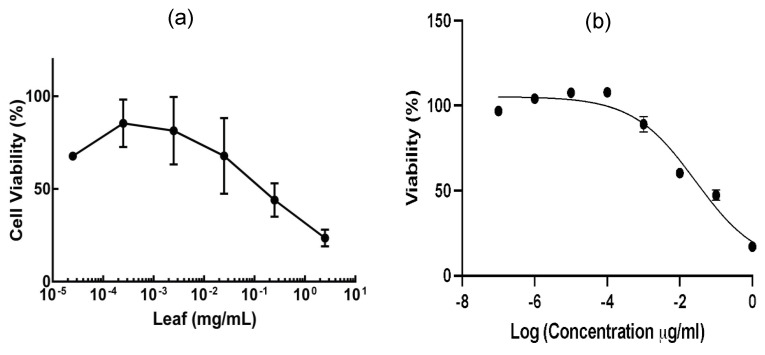
Dose response in cell viability of whole-leaf extract (**a**) and the L19 fraction (**b**) on DLD-1 cell line. The cells were exposed to serial dilution of whole-leaf extract and L19, in log-fold concentrations, using RPMI media. The cells were seeded at 1 × 10^4^ cells/well onto a 96-well tissue culture plate and allowed to attach overnight. The percentage of cell viability was determined after a 24 h incubation. Absorbance was measured at 490 nm, and cell viability was calculated relative to the untreated cells (n = 3) and their standard deviation.

Dose response experiments were performed to investigate if the induction of cell viability is dose-dependent in *R. crispus*. Whole-leaf extract as well as the L19 fraction exhibited a dose dependent response, as shown in Figure 3. The dose-dependent response was found to be consistent with the curve reported in the literature [26] and also similar to the dose curve for DOX in our experiment (Supplementary Figure S7). The characteristic sigmoidal curve, shown in Figure 3b, suggested that cell killing by L19 is carried out in a dose-dependent manner. The calculated IC50 value for the L19 fraction was 0.2 µg/mL.

### 3.4. Dose-Dependent Behavior of L19 Fraction on Caspase-Mediated Apoptosis

**Figure 4:**
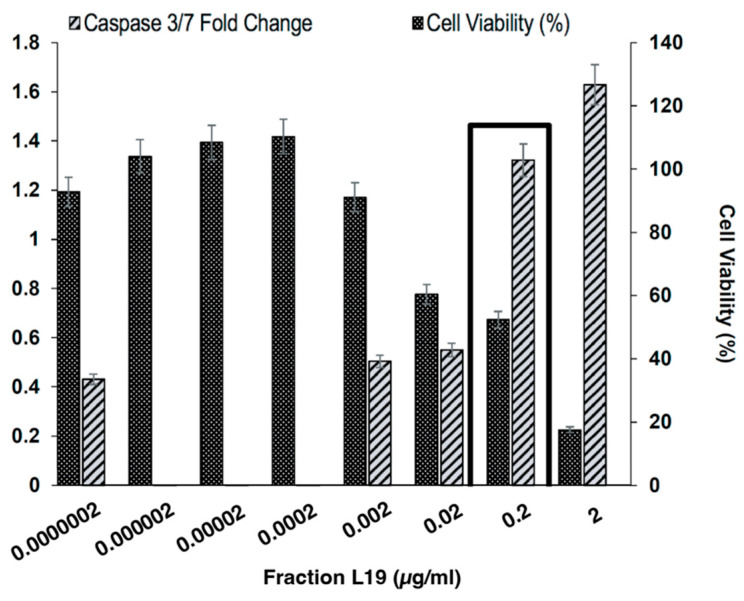
Caspase-3/7 fold change (left axis) and percentage of cell viability (right axis) in DLD-1 cells in response to different L19 concentrations.

Dose response experiments were performed to investigate if the apoptotic activity seen in caspase-3/7 fold change due to *R. crispus’* L19 fraction was dose-dependent. The *R. crispus* leaf fraction L19 exhibited high levels of caspase-3/7 expression when compared to the controls. The fold change in caspase-3/7 due to the L19 fraction was dose-dependent (Figure 4).

### 3.5. Time-Dependent Changes in CASP Gene Transcription Profile upon L19 Treatment

**Figure 5:**
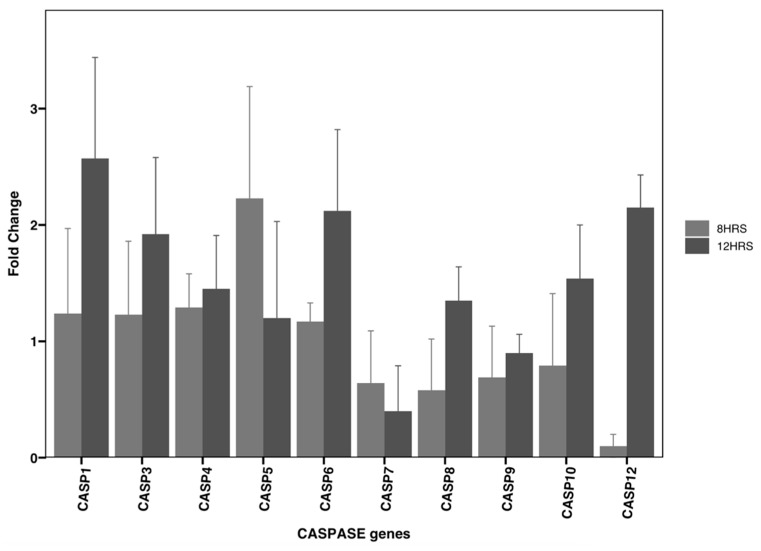
Changes in the relative mRNA transcription levels of CASP genes in DLD-1 cells upon treatment with L19 measured by an FC qRT-PCR assay. The scale bar represents the fold changes in the mRNA transcription levels when compared to the controls.

The RT-qPCR was performed for the L19 fraction with two different exposure times to assess the cellular activity of CASP induction-signaling cascades. It is known that DLD-1 cells present dysregulated caspase activity, which was assessed through the cellular mRNA transcription levels. The fold changes in CASP 5 were found to be two-fold higher over the course of the 8 h treatment measurement time points compared to the levels of other caspases (CASP 1, 3, 4, 6, 8, 10, and 12), while CASP 1 had its highest fold change during the 12 h treatment (Figure 5). The activation of different caspases at different times suggests that there is an activation of multiple pathways at different time points mediated through caspase cascades.

### 3.6. Changes in Apoptosis-Related Gene Transcription upon L19 Treatment

**Figure 6:**
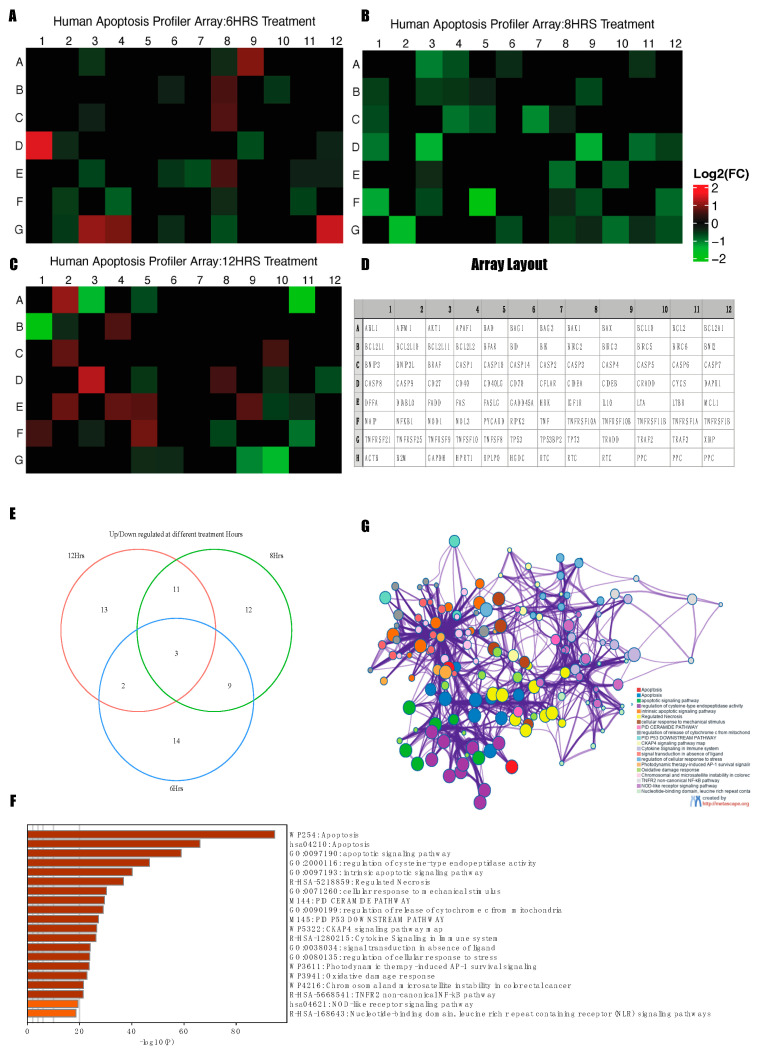
Human apoptosis RT^2^ profiler assay. Heatmap of the expression levels of genes (apoptosis and non-apoptosis genes) included on profiler array upon L19 treatment at 6 h (**A**), 8 h (**B**), and 12 h (**C**). Array layout of human apoptosis RT^2^ profiler assay (**D**). Venn-diagram of number of differentially expressed genes at three different time points (**E**). Enriched terms sorted by *p*-value (**F**) and protein–protein network analysis (**G**) for the differentially expressed genes using Metascape.

Gene transcription of eighty-four key genes important for the central mechanisms of cellular death by apoptosis, autophagy, and necrosis pathways were assessed with the human apoptosis RT2 profiler assay along with five housekeeping genes and controls. The mRNA transcription data were normalized to the Ct values based on two housekeeping genes (HK), Glyceraldehyde-3-phosphate dehydrogenase (GAPDH) and β-actin, as they showed no changes in response to L19 treatment when compared to the untreated cells at the variable time points. A greater than 1.5-fold change and less than 0.7 change in relative mRNA transcript synthesis was considered significant for this experiment. We observed distinct sets of apoptosis-related genes differentially expressed at the 6 h, 8 h, and 12 h treatment points (Figure 6A–C). Most apoptosis-related genes differentially expressed at the 8 h and 12 h treatment time points, whereas fourteen differentially expressed genes were found to be common at the 8 h and 12 h treatment time points (Figure 6E).

A gene ontology enrichment analysis was performed in the Metascape [27] platform, where differentially expressed significant genes were used to identify enriched terms using GO/KEGG terms, canonical pathways, and hall mark gene sets as the default settings on Metascape. The raw values for the annotation and enrichment results are in Supplementary Table S2. The enrichment analysis results confirm that the mitochondria-regulated apoptosis pathway is the most significantly enriched pathway among other pathways such as the immune pathway and the inflammatory pathway (Figure 6F). Additionally, a protein–protein interaction analysis showed that the enrichment terms and pathways related to intrinsic apoptosis, mitochondria-mediated apoptosis, necrosis, and oxidative damage response are majorly enriched networks (Figure 6G). The protein–protein interaction network highlights key biological processes like apoptosis and immune signaling, representing pathways such as cell death mechanisms and responses to stress and inflammation.

### 3.7. Microarray Analysis

**Figure 7:**
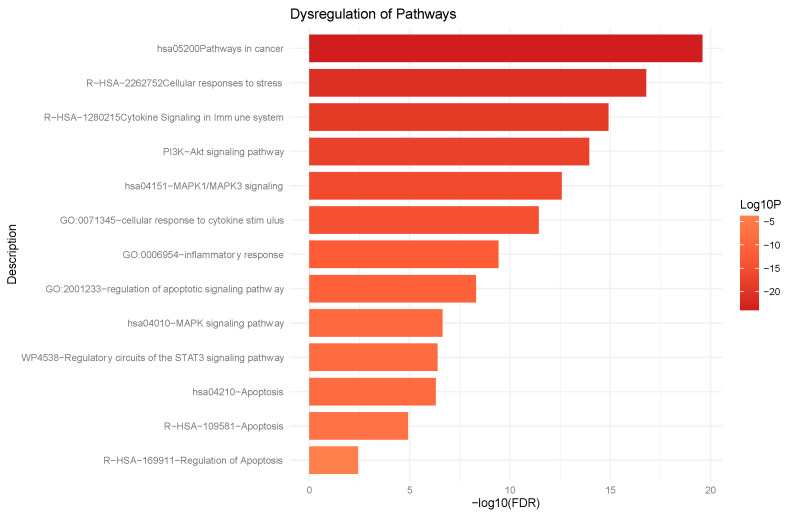
Gene ontology enrichment analysis using Metascape. Enriched pathways sorted by *p*-value.

Our microarray analysis indicated that, upon the treatment of DLD-1 cells with the L19 compound, several other cancer-related genes and their gene partners were differentially expressed. The list of genes with respective fold changes upon L19 fraction treatment measured at the 6 h, 8 h, and 12 h time points are summarized in Supplementary Table S3. These differentially expressed gene lists with significant *p*-values were used to perform a gene ontology enrichment analysis using Metascape. The enrichment analysis of these genes and gene partners shows gene sets that are involved in different pathways in cancer and in cellular responses to stress, cytokine signaling, the PI3K-Akt signaling pathway, the MAPK1/MAPK3 signaling pathway, inflammatory pathways, and the STAT3 pathways, as the most relevant pathways. Some of the most relevant pathways sorted by *p*-value can be seen in Figure 7. Other significant pathways such as the cell cycle pathway, the HIF-1 pathway, the metabolic pathway, and the EGF/EGFR signaling pathway were also significantly enriched. The enrichment scores obtained from the analysis are provided in Supplementary Table S4.

## 4. Discussion

The presence of diverse natural compounds in medicinal plants has attracted significant interest due to their potential anti-proliferative and antioxidant effects in cancer cells. Approximately 20% of known plants have been investigated for their pharmaceutical properties, as these natural products serve as lead compounds for drug development. This study sought to find novel water-soluble phytochemical compounds from *R. crispus* with enhanced chemotherapeutic potential. The targeting of water-soluble polar compounds ideally produces phytochemical extracts with potential targets that are also water-soluble and, therefore, readily absorbed into the body. This was accomplished through the medium-throughput screening of extract fractions from the roots and leaves of *Rumex crispus* plant tissues for anti-proliferative properties using human colorectal adenocarcinoma cells (DLD-1), and this process identified HPLC fraction L19, from the leaf fractions, as the most potent fraction.

A previous study has reported that a mono-tetrahydrofuran acetogenin from the seed of *Annona reticulata* showed anti-proliferative activity against the T24 bladder cell line by arresting the cell cycle in Bax- and caspase 3-related pathways [28]. Previous studies on *Rumex crispus* have reported its bioactivity as being antioxidant and cytotoxic in nature [29]. Another study has shown that, when tested in a human hepatoma cancer cell line (HepG2), *Rumex crispus* extract induces increased apoptosis through the activation of various caspase cascades and Bcl-2, Bax-mediated pathways [30]. Additional analytical and cancer bioassay studies on *Rumex crispus* by Cho et al. identified two organically soluble phytochemical constituents, chrysophanol and parietin, exhibiting quantifiable cytotoxicity with an LC50 of approximately 115 µg/mL [31]. Based on these findings, we know that *Rumex crispus* contains anti-proliferative and cytotoxic phytochemicals with the potential for cancer therapeutics and that our method produces similar results with aqueous whole-leaf extracts, while producing fractions which not only inhibit cancer cells but induce apoptotic cellular-signaling pathways. Furthermore, by turning on apoptotic signaling in cancer cells which are inherently resistant and enduring, our method provides a strategic pathway for improving an already-impaired genetic and epigenetic self-regulatory mechanism in cancer biology. At a minimum, by showing efficacy in increasing regulatory pathways of cellular self-governance, such as STAT3 and MAP kinases, our method indicates a differential transcription profile, from the untreated cells, demonstrating sensitization to the chemotherapeutic agents present in the phytochemical assays of *Rumex crispus*. Our result showed fraction L19 to present dose-dependent anti-proliferative activity and apoptotic activity through CASP mRNA transcripts and caspase-dependent pathways through the activation of CASP3 and CASP6. In comparison, Doxorubicin (DOX) induces apoptosis by arresting DNA replication, primarily through the inhibition of topoisomerase I and II [32]. However, the mechanisms by which *Rumex crispus* extracts facilitate apoptosis and the mechanisms behind its Absorption, Distribution, Metabolism, and Excretion (ADME) warrant further investigation. We also observed the activation of CASP1 along with changes in the transcription of other non-apoptotic genes, suggesting that the L19-induced cell cytotoxicity is achieved not purely via the apoptotic pathway but through a combination of both apoptotic and inflammatory pathways. The anti-proliferative and apoptotic property of the L19 fraction from this study could be further expanded in order to investigate other cancer types, using GC-MS-grade L19 pure compounds on different cancer and normal cell lines and seeking additional validations.

We showed that phytochemical extracts of *Rumex crispus* enhanced killing, apoptotic signaling, and cell death in DLD-1 adenocarcinoma cells, as indicated by the expression levels in our apoptosis assays and the mRNA transcription of apoptosis-related genes. Our MTS apoptosis assays for caspase-3/7 showed enhanced cell killing and indicated enhanced caspase production for extract fraction L19 in a dose-dependent manner. Our data suggest that the L19 fraction induces CASP8 expression; however, this result may be an effect seen with tumor necrosis factor-related apoptosis-inducing ligand (TRAIL) resistance in DLD-1 cells with enhanced caspase-8 mRNA transcription and reduced procaspase-8 production [33]. This could explain why caspase-3 and caspase-8 mRNA transcripts were expressed early in the experiments but not transcripts of CASP6, which has been shown to be downregulated by ARK5 expression and evade apoptosis [34,35]. As such, the over-transcription of CASP1 may be a response to dysregulated caspase family production as part of a PANoptosis cellular response.

## 5. Conclusions

We screened an extract from *Rumex crispus*’ root and leaves to investigate its anti-proliferative properties using the human colorectal adenocarcinoma cells (DLD-1) cancer line and identified fraction L19 from the leaf section as the most potent fraction. The L19 fraction activated caspase-dependent apoptosis and caused a significant amount of cytotoxicity to the cancer cells. This induction of apoptosis renders L19 a promising anticancer agent. L19 could serve as a strong lead compound for the synthesis of other novel potent analogues for different cancer types; it can also be further investigated for structural validation as well as in future animal studies to classify both the biochemical and cellular pathways involved in inducing a robust response in the chemotherapeutic-resistant cell line DLD-1.

## Data Availability

Information on the primers used in this study is available in Supplementary Table S1. The DEGS and Enrichment Scores (ES) are available in Supplementary Tables S2 and S4.

## Supplementary Materials

The following supporting information can be downloaded at https://www.mdpi.com/article/10.3390/life14010008/s1. Figure S1: SEER estimation of colorectal cancer; Figure S2: *Rumex crispus* plants collected from Nacogdoches, TX, USA; Figure S3: Cell viability screening for *R. crispus* root HPLC fractions; Figure S4: Cell viability screening for *R. crispus* leaf HPLC fractions; Figure S5: Dose response cell viability of DOX on DLD-1 cell line; Figure S6: A representative Brightfield image of DLD-1 cells at 200× magnification and ISO1600; Figure S7: Representative native agarose gel of the total RNA isolated from the untreated and treated cells after exposure to L19 for different exposure times; Table S1: ES for significantly enriched pathways among the DEGs between the L19-treated and control DLD-1 cells; Table S2: Summary of the significant DEGs between the L19-treated and control DLD-1 cells; Table S3: ES for significantly enriched pathways among the DEGs between the L19-treated and control DLD-1 cells from the microarray experiment; Table S4: Primers used for the RT-qPCR analysis.
