# Supplementary material for: Anti-Proliferative and Apoptotic Activities of Rumex crispus
Source: Life (Basel). 2023 Dec 20;14(1):8. doi: 10.3390/life14010008 (PMC10819952; doi:10.3390/life14010008)
Supplement: Supplementary file 1 [file life-14-00008-s001.zip › life-2718887-supplementary/life-2718887-supplementary/Supplementary/Supplementary Figures.docx]

**Supplementary Figures:**

S1**: (a) SEER estimation of Colorectal cancer as 4^th^ leading cause of cancer deaths as well as 4^th^ in estimated new cases. (b) SEER data indicates increasing trend in incidence rates of colorectal cancer in younger population of age less than 50.**


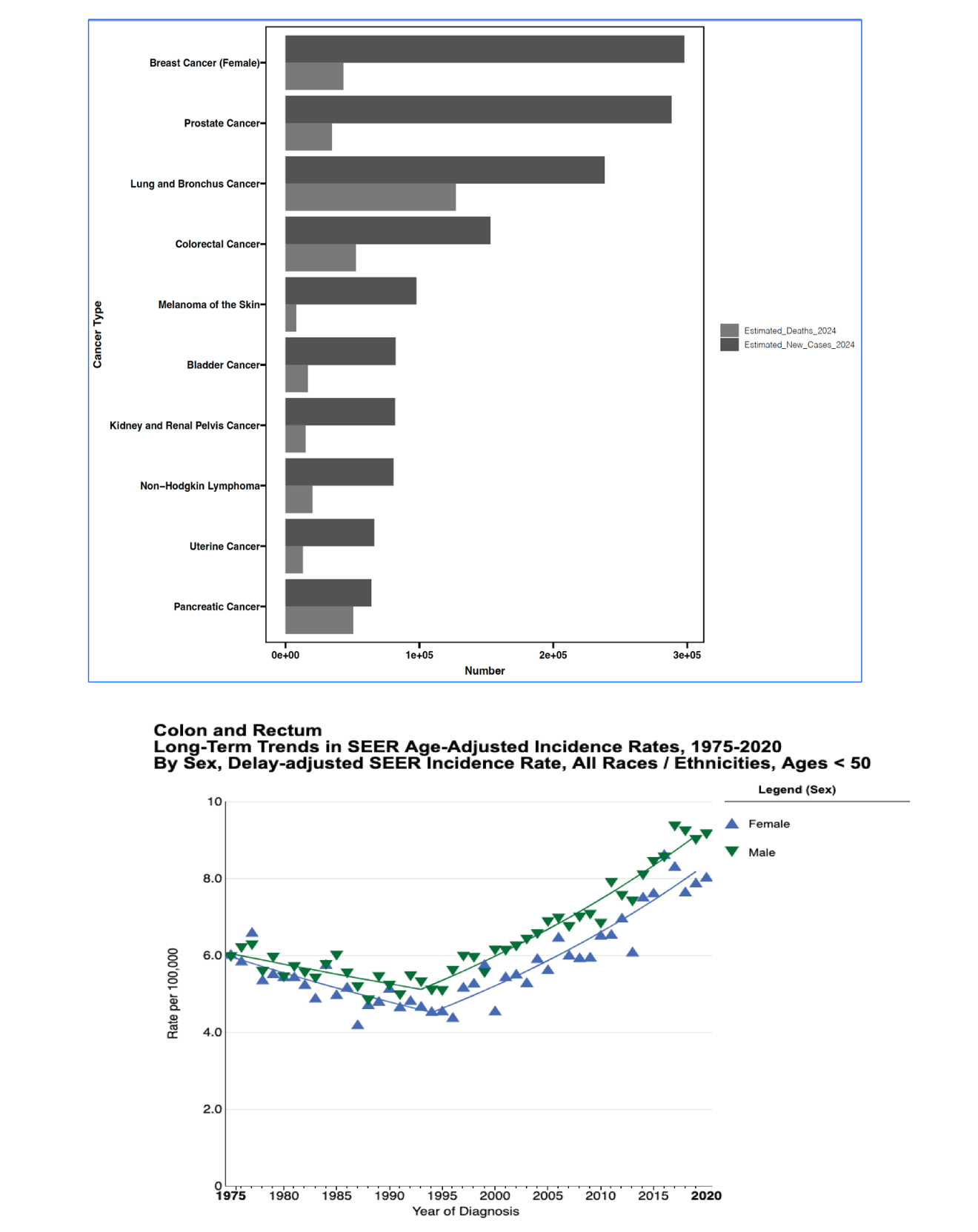


a

b

S2**:** *Rumex crispus* plants collected from Nacogdoches, Texas, USA (31.6024865 N, 94.5677874 W) in April 2014.


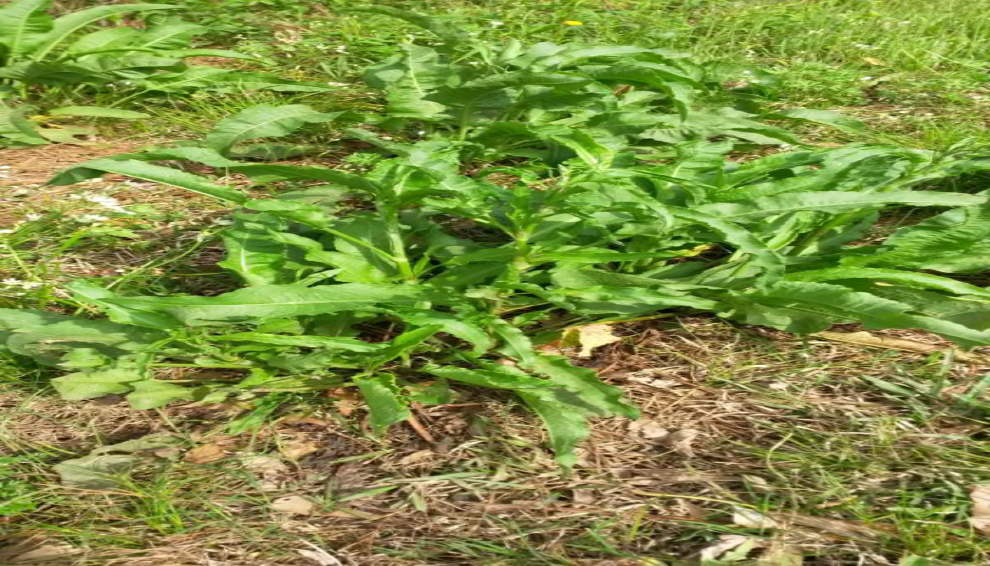


**S3:** A representative Brightfield image of DLD-1 cells at 200 x magnification and ISO1600.


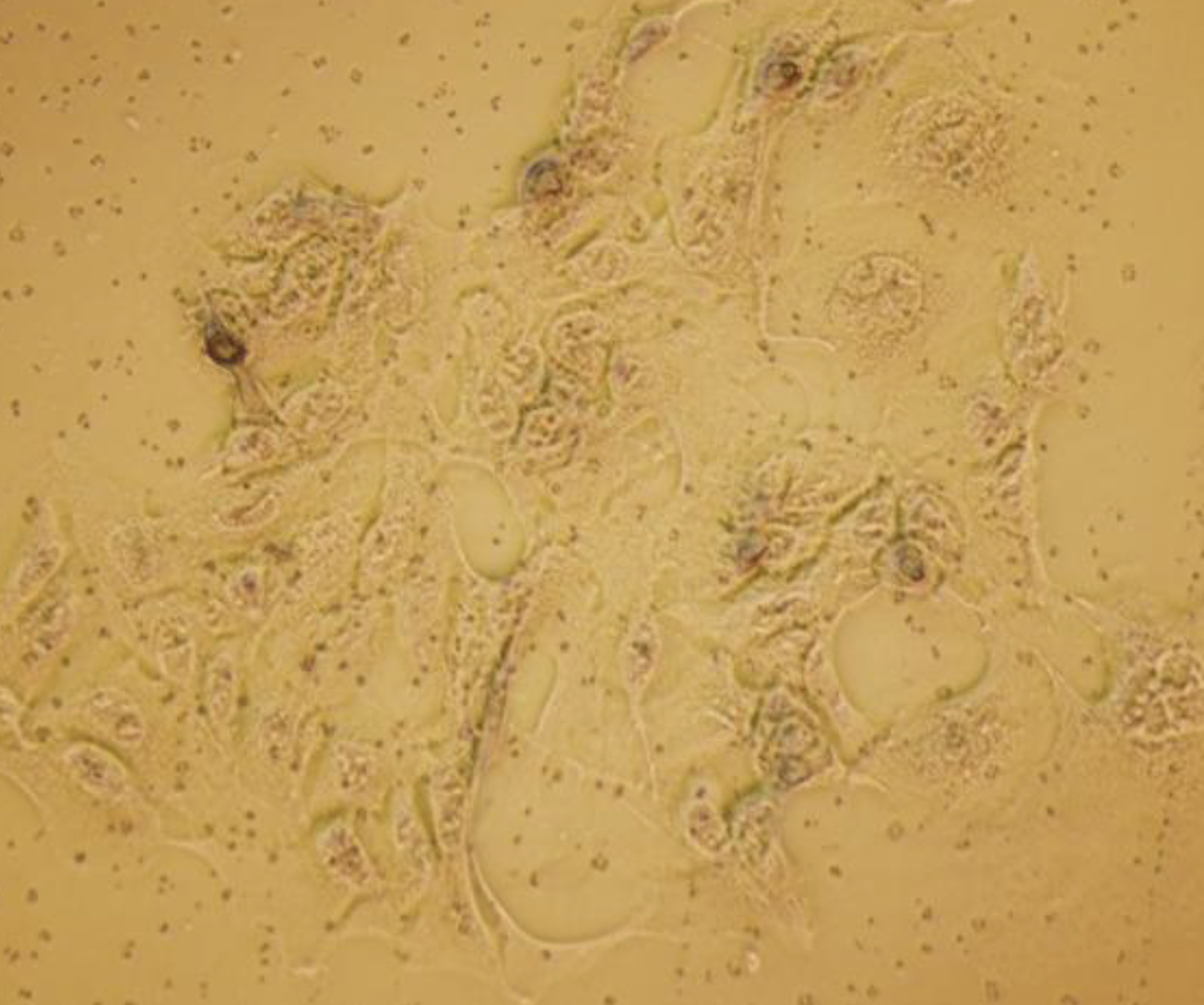


**S4:** Representative native agarose gel of total RNA isolated from the untreated and treated cells after exposure to L19 for different exposure times. The quality of total RNA was determined by examining the 28S and 18S rRNA on a 1% native agarose gel. Total RNA for two different samples isolated from untreated cells, UT1 and UT2 are shown in lanes 1 and 2. Lane 3 contains the All Purpose HI-LO^TM^ DNA marker (50–10,000 bp) and the lanes labeled T1 and T2 contain total RNA isolated from treated cells. Each lane contained 5 µL total RNA and 5 µL bromophenol blue loading dye.


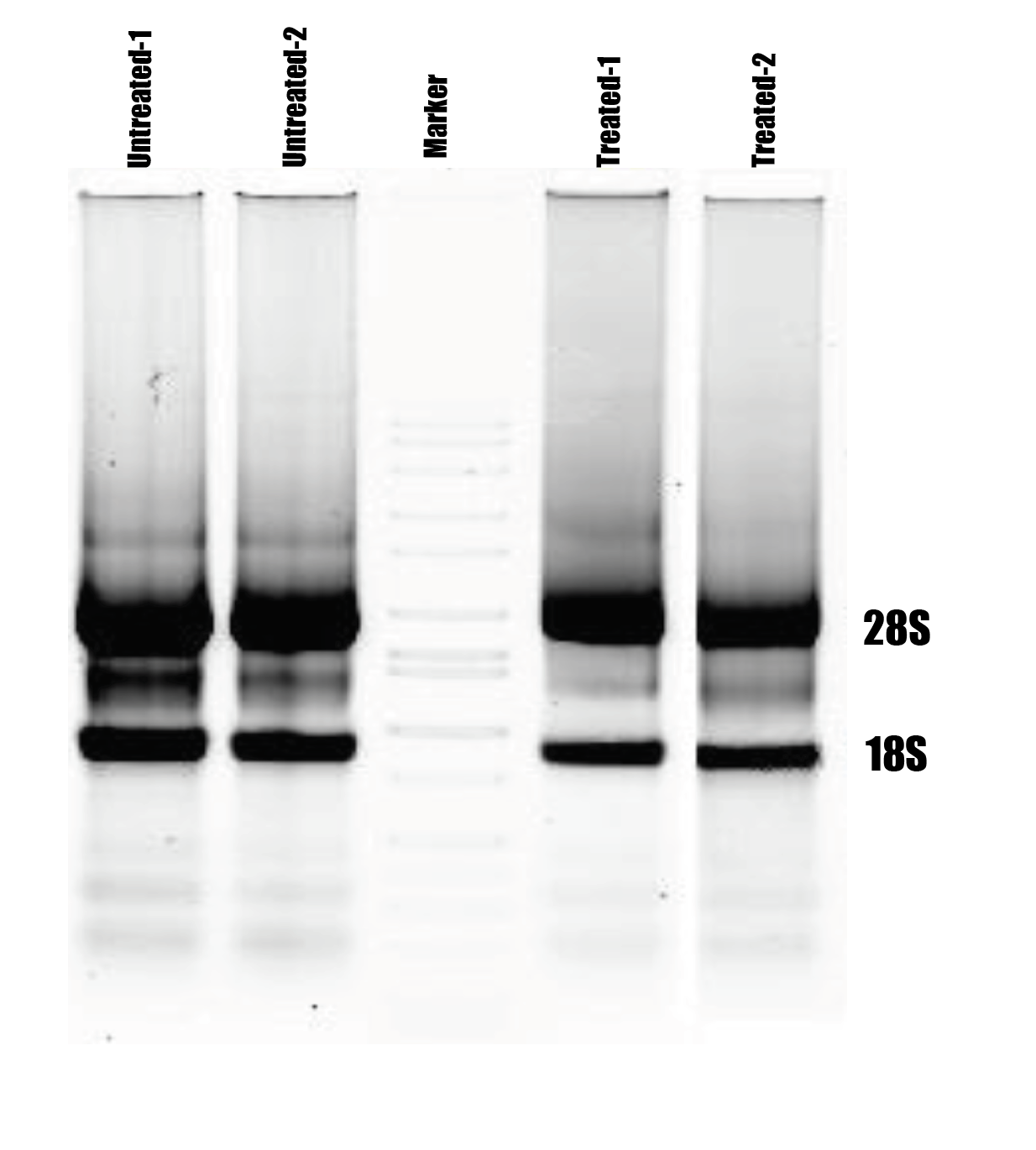


S5: Cell viability screening *for* R. crispus root HPLC fractions


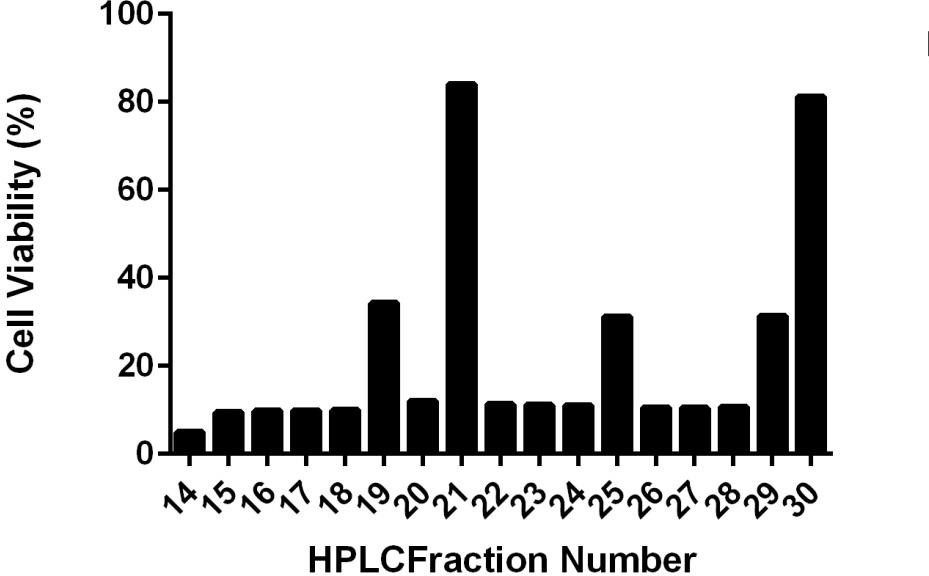


Initial screening for cell viability on selected HPLC root extracts from fraction 14 to fraction 30. Lyophilized HPLC fractions were resuspended in 100 μL RPMI plus and cell viability assays were performed in single wells (N=1) dosing 1X10^4^ DLD-1 cells.

S6: Cell viability screening *for* R. crispus leaf HPLC fractions


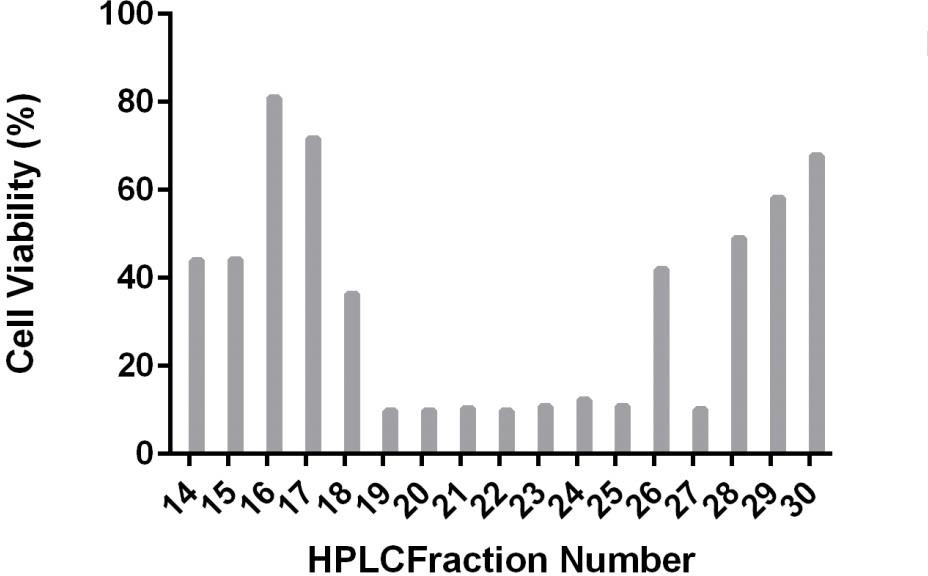


Initial screening for cell viability on selected HPLC leaf extracts from fraction 14 to fraction 30. Lyophilized HPLC fractions were resuspended in 100 μL RPMI plus and cell viability assays were performed in single wells (N=1) dosing 1X10^4^ DLD-1 cells for initial screening purpose. The figure is generated using GraphPad Prism software.

S7: Dose response cell viability of DOX on DLD-1 Cell line


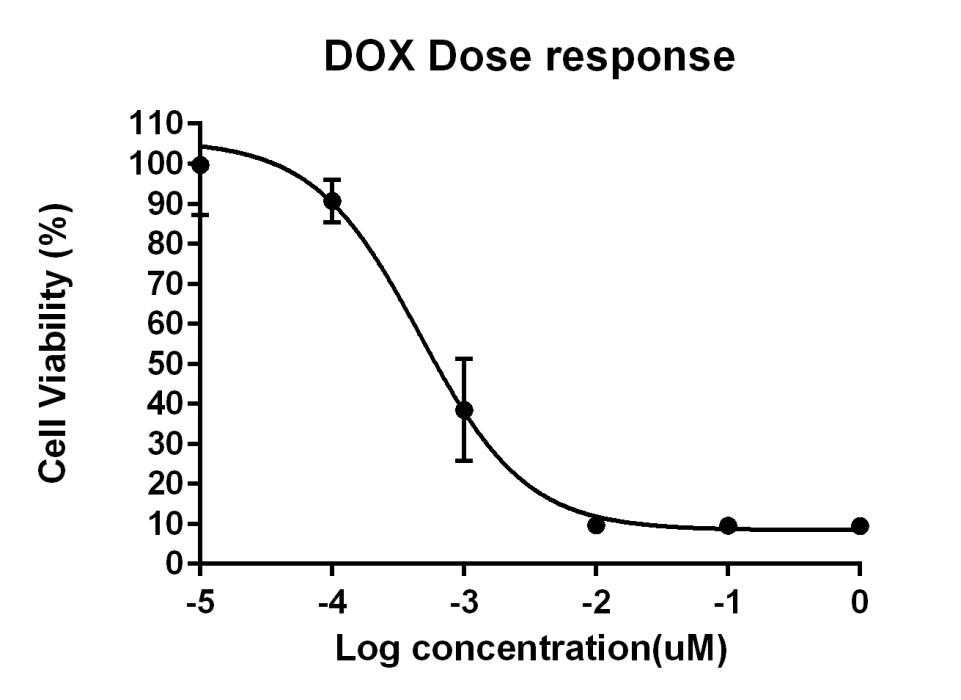


Dose response cell viability of DOX on DLD-1 Cell line. A serial dilution of 50µM doxorubicin hydrochloride was done in log fold using RPMI plus to result to in 100µL volume in each well. Cells were seeded at 1x10^4^cells/well onto a 96 well tissue culture plate and allowed to attach overnight. Triplicates of each dilution were done. After 24 hours incubation percent viability was determined using CellTiter 96® AQueous One Solution Reagent (Promega Corp., Madison, WI, USA). Absorbance was measured at 490nm and cell viability was calculated relative to untreated cells as 100% viable. Error bars represent standard deviation between the triplicates.
